# Rethinking the Epigenetic Framework to Unravel the Molecular Pathology of Schizophrenia

**DOI:** 10.3390/ijms18040790

**Published:** 2017-04-07

**Authors:** Ariel Cariaga-Martinez, Raúl Alelú-Paz

**Affiliations:** 1 Laboratory for Neuroscience of Mental Disorders “Elena Pessino”, Canis Majoris Foundation, Madrid Science Park, 28049 Madrid, Spain; acariaga@canismajoris.es or ariel.cariaga@uah.es; 2 Department of Medicine and Medical Specialties, Faculty of Medicine, University of Alcalá, Universitary Campus, A2-Km 33.6, Alcalá de Henares, 28805 Madrid, Spain; 3 Department of Psychiatry, Ramón y Cajal Hospital, IRYCIS, Ctra Colmenar Km 9.1, 28034 Madrid, Spain

**Keywords:** schizophrenia, human brain, epigenetic, neuroepigenome, molecular pathology

## Abstract

Schizophrenia is a complex mental disorder whose causes are still far from being known. Although researchers have focused on genetic or environmental contributions to the disease, we still lack a scientific framework that joins molecular and clinical findings. Epigenetic can explain how environmental variables may affect gene expression without modifying the DNA sequence. In fact, neuroepigenomics represents an effort to unify the research available on the molecular pathology of mental diseases, which has been carried out through several approaches ranging from interrogating single DNA methylation events and hydroxymethylation patterns, to epigenome-wide association studies, as well as studying post-translational modifications of histones, or nucleosomal positioning. The high dependence on tissues with epigenetic marks compels scientists to refine their sampling procedures, and in this review, we will focus on findings obtained from brain tissue. Despite our efforts, we still need to refine our hypothesis generation process to obtain real knowledge from a neuroepigenomic framework, to avoid the creation of more noise on this innovative point of view; this may help us to definitively unravel the molecular pathology of severe mental illnesses, such as schizophrenia.

## 1. Introduction

Schizophrenia is a complex disease characterized by the heterogeneous presence of cognitive symptoms affecting perception, thought, attention, memory and emotion. Several complex factors including environmental, social and genetic background contribute to its development.

We have more data about schizophrenia than ever before, yet its underlying causes are still unknown [1,2]. The development of a plethora of innovative techniques that lead from clinical research to the laboratory, from genes to disease, has resulted in the accumulation of massive quantities of data in the quest to unravel the origins of this condition. However, we are no nearer to understanding the disorder than we were a decade ago.

It is clear that the concordance rates of schizophrenia for monozygotic twins is about 40–50%, and inheritability is around 80% [3,4], highlighting the significant genetic contribution to the development of this disease. However, this relationship has been interpreted in the last decades as genetics alone being the cause, but this cannot fully explain its etiology and pathogenesis [5,6]. For instance, by using genomic-wide association studies (GWAS), researchers are trying to understand the association of genetic polymorphism with schizophrenia. However, this technique is extremely inefficient. Using the same type of samples, with the same diagnosis, different groups have obtained different new schizophrenia gene lists, and in the absence of any single widely accepted one, the general solution is to increase the sample size in subsequent studies. This has not solved the issue, although at least, these studies allow us to reach several conclusions: Firstly, GWAS require thousands of samples and several steps of data “cleaning”; furthermore, the “rarer” the polymorphism, the more complicated are the statistical studies required in order to avoid false positives [7,8]. Secondly, in GWAS studies we only get a ratio, without knowing what this ratio means biologically. Thirdly, GWAS data cleaning is extremely reductionist, and from thousands of possibly associated polymorphisms we usually obtain only one or two significant ones; if disease development in fact depends upon the contribution of several genetic polymorphisms we will not be able to assess it by using the GWAS technique [9]. Furthermore, in contrast with cancer, where cell homogeneity is relatively constant (although not absolutely [10]), the neurons and brain share, in the words of Santiago Ramón y Cajal, a form-function combination that is hardly adaptable to the GWAS hypotheses. Recently, contributing to this disordered situation, Evrony et al., using single-cell DNA sequencing, demonstrated the existence of somatic mutations in different neurons; this means that, even inside a single brain, the genetic background is highly variable, so sampling methods must be extremely refined in order to get reproducible results [11].

While we try to understand the genomic architecture of the disorder, what about environmental factors?

The full onset of schizophrenia is associated with environmental roots such as early life adversity, growing up in an urban environment, minority group membership, and drug abuse, affecting to some extent the developing “social” brain during sensitive periods [12]. Up to now, these environmental contributions have been difficult to assess through molecular biology techniques. For overcoming this issue, researchers are focusing on epigenetics, biological mechanisms that allow gene regulation without making changes to the DNA sequence. These mechanisms include DNA methylation, histone post-translational modifications, and miRNA and nucleosome positioning. The interplay between these mechanisms dictates the final biological outcome [13].

This area of research has been successfully applied in cancer, and its findings have enabled diagnostic tools, drug discovery and innovative treatments to be developed [14]. However, using this approach with psychiatry, current research only counts on fragmented information, focusing on DNA methylation as the main finding, with minimal interest in the interplay with other epigenetic mechanisms. Given the current outlook, and with the current lack of information, we are unable to get an adequate frame of knowledge of the actual epigenetic contribution to mental disorder development.

With regard to the application of epigenetic approaches to study the brain and its related disorders, the term “Neuroepigenome” was coined [15]. Neuroepigenomics attempts to build a frame of knowledge that harmonizes all the information on the genome, as a layer that also includes three-dimensional information, and interactions between biological mechanisms, mainly through regulation of gene expression. Methylation itself (along with other DNA modifications) is not sufficient to fully understand the putative role of epigenetics in mental disorder development [16].

In this review, we will collect and summarize the current efforts to understand the molecular pathology of schizophrenia, through three main experimental approaches: (a) DNA methylation in brain samples (mainly assessed by Epigenome-Wide Association Studies or EWAS) and other DNA chemical modifications; (b) histone post-translational modifications; and (c) findings on the role of nucleosome remodeling in schizophrenia.

## 2. DNA Methylation

DNA methylation specifically refers to cytosine (C) methylation. Although adenine methylation might play a role as an epigenetic marker, it is still under consideration [17] and was only recently demonstrated to be present in embryonic stem cells [18]. In this review we will focus on cytosine methylation. This chemical modification implies the addition of a methyl group to the 5′ position of the pyrimidine base, to generate 5′-methyl-cytosine (5′mC). When this modification occurs mainly in areas with high concentration of guanine (G), it defines the so-called CpG islands. These islands are particularly enriched along gene promoters, so the pattern of cytosine methylation may affect the gene expression, either by activating or repressing transcription [19]. It is generally accepted that promoter hypomethylation represents an activating signal, while hypermethylation represents a repressive mark [20]. However, the biological outcome of DNA methylation is more complex than this, especially with the discovery of methylation changes in gene bodies, as well as non-coding regions and RNAs [21,22,23].

Regarding the relationship between the molecular pathology of schizophrenia and gene methylation status, initial reports focused on single gene or promoters. Although in this work we will not assess all the reports centered on single genes, we will indicate some key findings in gene methylation in the context of neuronal biochemistry, obtained by using brain tissue. Although these were pioneer studies, they became obsolete after the development of EWAS. This will be discussed later in the review.

The first studies on the role of promoter methylation and schizophrenia onset were focused on the epigenetic regulation of reelin expression from the *RELN* gene. This extracellular matrix protein plays a role in the formation of synaptic circuits [24], and its expression was previously related to schizophrenia development [25]. Several research groups were interested in the regulation of reelin expression by methylation changes in its promoter [26], and showed that this mechanism may be involved in the observed decrease in reelin observed in psychiatric patients [27,28,29]. 

Although the observation above suggested an advance in the understanding of the role of DNA methylation in brain tissue, several methodological handicaps (for instance, the usage of wide-spectrum demethylation drugs to revert the observed phenotypes) prevented the elucidation of the concrete role of reelin in psychiatric disorders. Although Abdolmaleky et al. found some differences in methylation along the *RELN* gene (not in the promoter region) between schizophrenic and healthy individuals [28], they did not consider the tissue heterogeneity and the statistical power of the study. Further, as Veldic et al. recognized, it is not possible to know whether observed changes in reelin expression are a molecular cause or a consequence, despite its possible role in GABAergic neuron impairment observed in the schizophrenic brain [30]. To date, the role of reelin is still under investigation for several mental illnesses [31]. Powerful detection tools and careful sample selection are required in order to correctly understand the molecular implications of methylation at a single gene locus, either in development, or as a risk factor for mental illness.

The interaction between gene methylation status and several other candidate genes was under study in the schizophrenic brain, patients or cohorts. For instance, it was observed that the human brain-derived neurotrophic factor (*BDNF*) gene might undergo epigenetic regulation [32], and it was also observed that there was a reduction of BDNF levels in schizophrenic brain tissues [33,34]. In conjunction, it was proposed that methylation changes might be responsible for the changes in BDNF levels in schizophrenic brain tissues. However, this hypothesis is hard to reproduce and subsequent findings have shown positive [35] and negative [36,37] correlations associated with the methylation status of *BDNF* and its expression levels.

All this contradictory information may be due to the use of surrogate tissues (especially blood samples) and the inherently complex organization of the *BDNF* that masks its definitive role in the molecular pathology of complex mental illness. In summary, these observations and studies need careful analysis in order to assign a role to *BDNF* in the molecular pathology of schizophrenia.

The methylation of the catechol-*O*-methyltransferase (*COMT*) gene promoter is also a candidate for the molecular pathology of schizophrenia. *COMT* genes encompass two promoters that generate a short soluble isoform (*S-COMT*) and a membrane bound isoform (*MB-COMT*). The first description of the methylation pattern of *S-COMT* promoter was reported by Murphy et al., although this pattern was assessed in only one schizophrenic patient [38]. A later report stated that *MB-COMT* promoter hypomethylation was a major risk factor for schizophrenia, although this report did not consider the cell heterogeneity of brain tissue, as it only centered on the anatomic area, and methylation status was only assessed by using methylation-specific polymerase chain reaction (MSP) [39]. Conversely, Dempster et al. did not find differences in *COMT* methylation status in cerebellum tissue of schizophrenic patients [40], highlighting the tissue specificity of putative epigenetic marks.

Furthermore, a more recent report indicates that *MB-COMT* promoter methylation might be related to working-memory processing in schizophrenia patients [41]. However, these data were obtained from blood samples, so methylation analysis and correlations should be carefully taken. As we can see, surrogate tissues are common resources for these reports, hence contradictory data is common.

The methylation pattern of other single genes was also assessed. For instance, Iwamoto et al. [42] observed differences in *SOX10* gene methylation when they assessed 23 schizophrenic brain samples. Interestingly, these methylation changes might be related to oligodendrocyte dysfunction. Also, Murphy et al. described the methylation patter of *SYNII* gene in a distal CpG island, in a single schizophrenic patient [43], without finding differences with regard to healthy controls.

Another single gene under study is the 5-HT2A receptor gene (*5-HT2AR*), a serotonin receptor. The differential expression of the C(102)T polymorphism of this receptor was observed in schizophrenic brain tissue, where “C” allele was mainly expressed [44]. Polesskaya et al. showed that this allele had two additional CpG sites that were not present in the “T” allele, and that those CpG sites were under epigenetic regulation: hypermethylation of these sites led to a higher expression of *5-HT2AR* [45]. A later report assessing 35 brain samples of schizophrenic patients, shed more light on this issue by the finding that the promoter of this gene is also under epigenetic regulation [46].

It became clear at least a decade ago that methylation changes in a single gene are not enough to enable us to understand a complex illness such as schizophrenia, and first enzymatic restriction and array assays were developed. Through these methods, Mills et al. found, by using 35 brain samples, stronger evidence that the methylation of *BDNF* in schizophrenic brains was correlated to the presence of non-synonymous single-nucleotide polymorphisms (SNP) that were previously associated with schizophrenia [47], establishing a genetic-epigenetic bridge in mental illness research.

Given the aforementioned complexity, EWAS arose as an option to explore several gene regions at the same time. Currently EWAS can assess around 800,000 sequences susceptible to methylation, bringing us a comprehensive map of methylation along the whole genome [48]. This technique also allows assessing of methylation at “non-CpG” sites, whose functions are far from established [49]; these we will briefly review below.

EWAS are a unique tool for understanding the role of DNA methylation in the molecular pathology of schizophrenia. In Table 1, we summarize the main findings obtained using this technique to research the schizophrenic brain and cerebellum tissue over the last five years. Please note that summarized EWAS only encompass those conducted using brain samples. As mentioned above, epigenetic marks are tissue-specific, so information from surrogate tissues is not completely reliable for understanding the molecular pathology of mental illness [2].

Several tools were applied in these studies. For instance, Wokner et al. [50] assessed around 485,000 CpG sites, finding 2929 differentially methylated positions (DMPs) among healthy and schizophrenic patients. They observed that 1291 DMPs were located in CpG islands, and 817 were in promoter regions. In a new EWAS performed in 2015 [54], they also added a publicly available dataset to their initial study and found consistent DMPs upstream or in exons of six candidate genes *CERS3*, *DPPA5*, *REC8*, *PRDM9*, *LY6G5C* and *DDX43*. On the other hand, Chen et al. [51] assessed around 70,000 CpG sites in cerebellum tissue, finding 204 differentially methylated sites in schizophrenic patients. However, only four of these DMP CpG were significantly correlated with differential expression of genes (*PIK3R1*, *BTN3A3*, *NHLH1* and *SLC16A7*). This study highlights the tissue specificity of methylation and indicates that changes in methylation status are not always directly correlated to changes in protein expression.

Pidsley et al. [52] were more ambitious and assessed not only methylation of brain and cerebellum tissue, but also fetal brain tissue, in order to gain insights into the role of methylation changes during brain development. They found some evidence of the early neurodevelopmental component, and suggested that epigenetic mechanisms may mediate these effects. However, the more remarkable DMPs were found in prefrontal cortex tissue, including DMPs in genes that were previously related to the genetic background of schizophrenic brain as *CACNA1G* [56] and *HTR5A* [57].

Numata et al. [53] used a large cohort of brain samples, finding 107 DMPs, although they assessed a lower number of CpG sites (around 27,000). Of these, 79 showed hypermethylation, while 28 showed significantly reduced methylation. This study was extended by adding a quantitative trait loci analysis (mQTL) and genome-wide transcriptional profiling data of the prefrontal cortex tissue from the same subjects, finding only 86 correlations between methylation changes and gene expression [53].

Finally, a recent EWAS was performed by Alelú-Paz et al. [55] in several areas of the brain (including prefrontal cortex, hippocampus and anterior cingulate cortex), to assess the role of methylation changes in cognitive impairment of schizophrenic patients. In this pilot study, they found 139 DMPs and a global hypermethylation pattern in all the structures analyzed. Furthermore, in highly cognitive impaired schizophrenic patients, DMPs were different according to the assessed brain area, when compared to healthy controls [55].

Although these are significant contributions, none of these studies considered the cell heterogeneity of brain tissue. In general, it seems that epigenetic changes tend to concentrate around loci related to neuron development, cytoskeletal reorganization and neurotransmission. However, there is not enough information or clear enough trends to indicate specific signaling pathways. In addition, EWAS are unable to link correlations between epigenetic marks and biological outcomes: the most we can get from isolated EWAS is that some epigenetic marks are different between healthy and schizophrenic brains. But, what does this mean in relation to understanding the molecular pathology of this disorder? Despite the vast amounts of collected data, we need to be more precise in our sampling procedures and more specific in our hypotheses development, in order to gain benefits from these technologies and techniques.

Another putative epigenetic mark is non-CG methylation, or methylation of cytosine adjacent to bases other than guanosine. In general, this modification is related to gene repression [58], and is highly frequent in brain tissue [59]; however its role in schizophrenia development remains unknown. An early report by Inoue et al. indicates that non-CG methylation might be involved in transcription factor binding to the synaptotagmin XI (*SYT11*) gene promoter [60]. This gene is also related to schizophrenia onset [61]. However, this modification is still poorly explored and needs closer study in order to clarify its role in mental disorder development.

Finally, despite all the generated data, there are several confounders that may obscure the potential role of methylation changes in schizophrenia development and progression. Although smoking is one known variable [62,63,64], several drugs used during psychosis treatment also directly affect the gene methylation status. For instance, valproate acts as a histone deacetylase inhibitor, and in combination with clozapine (or sulpiride), may cause chromatin remodeling in schizophrenic brains [65]. On the other hand, clinically relevant doses of haloperidol or olanzapine did not increase demethylation of *RELN* or *GAD67* promoters [66]. However, we still lack information about other promoters in this regard.

In summary, DNA methylation studies (either through initial MSP, or through more complex assays such as EWAS) represent an innovative point of view in researching complex mental illnesses. However, actions such as reducing the “neuroepigenome” term to genes and environment interactions, or confounding work such as underpowered studies or by using surrogate tissues, continue to cause confusion within the field of mental illness studies. Finally, in order to better understand the molecular pathology of complex mental illness such as schizophrenia, we should avoid comparison of cases and techniques with other illness, especially that of cancer. Mental disorders are challenging, but with a careful process of hypotheses generation, we may be able to obtain more valuable knowledge that may directly impact the treatment and wellbeing of schizophrenic patients.

## 3. DNA Hydroxymethylation

This chemical modification refers to the addition of a hydroxymethyl group to cytosine, generating 5-hydroxymethyl-2′-deoxycytidine (5′hmC), which is implicated in regulating gene expression in mammals, as is genomic 5mC [67,68]. In moderately or highly transcribed genes, 5′hmC is generally depleted closer to the transcription start site (TSS) and enriched about 0.5–2 kb upstream and downstream, just at the borders of promoters, whilst poorly transcribed and untranscribed genes have a peak of 5′hmC at TSS itself [67].

The global levels of this epigenetic mark are much higher than in other tissues [69,70] both in CpG islands and intergenic regions [71,72,73]. Surprisingly, this modification is not sufficiently considered in the current epigenetic approach applied to understand the molecular pathology of schizophrenia, although some reports indicate that 5′hmC might be involved in key neural activities that lead to learning and memory formation [74,75]. To the best of our knowledge, in the literature, we only found one paper on this topic (PubMed: 5hmc [All Fields] AND (“schizophrenia” [MeSH Terms] OR “schizophrenia” [All Fields])). The aforementioned paper suggests that TET1 is markedly increased in the parietal cortex of psychotic patients and this increase is associated with an increase of 5hmC level at genomic DNA, and with specific increases of 5hmC levels at *GAD67* and *BDNF4* promoters in proximity of their TSS [76]. TET1 may have an important role in triggering neuronal activity-induced enzymatic removal of 5mC in mammalian brain, by hydroxylating 5mC residues at CpG-rich gene bodies, or at TSS of specific promoters [76].

## 4. Histone Modifications

As with several proteins, histones are also able to undergo post-translational modifications, such as acetylations, methylations, phosphorylations, SUMOylations and others [77,78]. But, what is the role of these modifications in gene expression? These modifications regulate the accessibility of the transcription machinery to DNA through histone acetyltransferases (HATs), deacetylases (HDACs), methyltransferases and demethylases, which are involved in histone acetylation, deacetylation, methylation and demethylation respectively, although there is a complex scheme of enzymes responsible for histone post-translational modifications, and its list may not be completed yet [79,80]. In this sense, euchromatin (actively transcribed chromatin) is characterized by the presence of high levels of acetylated, phosphorylated and trimethylated histones (H3K4, H3K27, H4K20, H3S10) while heterochromatin (transcriptionally inactive) shows low levels of acetylation but high levels of methylation of histones (H3K9, H3R17, H3K27 and H4K20) [81].

Although the first histone modifier enzyme was described twenty years ago [82] histone modification is currently recognized as a key epigenetic mechanism. Interest in histone modification arose in the field of Psychiatry around a decade ago, although earlier reports did not focus on the role of HDAC as an epigenetic mechanism, but on the genetic role of the enzyme as a potential diagnostic marker [83]. For DNA hydroxymethylation, there are few works on understanding the relationship between histone modifications and schizophrenia. Recently, histone methylation (common variants) has been implicated as a general biological process involved in schizophrenia pathogenesis [84,85,86], specifically *SETD1A*, a component of a histone methyltransferase complex that produces H3K4me, H3K4me2 and H3K4me3. According to the results, all heterozygous carriers of *SETD1A* are rare loss-of-function variants that satisfied the full diagnostic criteria for schizophrenia, including classic positive symptoms, and this classes *SETD1A* as a new gene for susceptibility to the disease [85,86].

Other altered histone modifications associated to schizophrenia include: (1) *PRDM9* hypermethylation, a gene that encodes a histone methyltransferase that specifically trimethylates H3K4, playing a central role in the transcriptional activation of genes during early meiotic prophase [54], (2) significant disruption of the ubiquitinylation, SUMOylation, NEDD8ylation, and Ufmylation systems in the schizophrenic superior temporal gyrus and, finally, a decrease in the expression of *PIAS3*, an E3 ligase which catalyzes the covalent attachment of a SUMO protein to specific target substrates. It directly binds to several transcription factors and either blocks or enhances their activity [87], (3) changes have been reported in prefrontal cortex in the chromatin surrounding *GAD1* promoter, accompanied by a decrease in its expression and, therefore, affecting the production of GABA, the main inhibitory neurotransmitter of the central nervous system [88], (4) in the same structure, H3R17me is associated with the down-regulation of *CYTOC/CYC1*, *MDH* and *OAT*, all being related to metabolic pathways reported for schizophrenia-like ornithine-polyamine metabolism, mitochondrial electron transport and the tricarboxylic acid cycle [89], (5) hypoacetylation of H3K9K14 at the promoter regions of *UGT8* (related to the biosynthesis of a key component of the myelin membrane of the central nervous system), *HTR2C* (serotonin receptor signaling), *GAD1*, *TOMM70A* (mitochondrial function), *MBP* (encoding the major constituent of the myelin sheath of oligodendrocytes and Schwann cells in the nervous system) and *PPM1E* (protein phosphatase, Mg^2+^/Mn^2+^ dependent 1E) [90,91], (6) decreased expression of *HDAC3* in the temporal cortex, associated to oligodendrocyte differentiation alterations [85,92] and upregulation of *TET1* and *HDAC1* in the hippocampus and prefrontal cortex [93,94,95], (7) increased mRNA levels of *EHMT1*, *EHMT2* and *SETDB1* (involved in the repression of transcription through H3K9me) [96] and, finally, (8) decreased levels of *HDAC2* in schizophrenic dorsolateral prefrontal cortex samples [97].

## 5. Nucleosome Remodeling

Nucleosomes represent the basic sub-unit of chromatin, which consists of 146 DNA base pairs spooled around a histone octamer in 1.65 left-handed superhelical turns [98]. These structures, when located in promoters and enhancers, are associated with gene repression, although they can also facilitate transcription by bringing distal regulatory sites into proximity, so that their positioning has either a repressive or an activating effect on gene expression [99,100]. Although nucleosome remodeling has rarely been explored in normal and schizophrenic human brains, one nuclear enzyme complex has significant effects on neuron-specific gene expression throughout development and adulthood [101]. Mutations in this complex, called the neuron-specific Brg1/hBrm Associated Factor (*nBAF*), have been linked to schizophrenia, specifically mutations in *SMARCA2* [102,103].

## 6. Conclusions

Epigenetics represents a rapidly growing and promising field that is expected to lead to the unravelling of the molecular mechanisms that are characteristic of schizophrenia. However, the complex picture presented in this paper reflects the important contradictions that we find when we face the study of such a complex disease. Unanswered questions still remain, and others arise as a result of study on the integration of different epigenetic marks. We still need to understand whether epimutations reflect long-lasting and sustained defects in the regulation of gene expression, whether different biological pathways that lead to schizophrenic symptoms could occur simultaneously as independent or as interdependent processes, and clarify when an epigenetic alteration is a quirk or a consequence of the disease. We know that we are far from being able to respond satisfactorily to all these issues, but we are not starting from scratch: it is necessary to introduce more clever and honest research strategies, which means, among other things, the use of human brain samples in the study of the epigenetics of schizophrenia, avoiding the use of surrogate tissues (i.e., peripheral blood or saliva samples), whose relevance as potential biomarkers is highly questionable as epigenetics is specific for every tissue and every single cell type [2,104]. Lack of reliability and validity will lead us to perpetuate uncertainty and confusion which is characteristic of the “publish or perish” philosophy. For this reason, in this review we decided not to include a significant number of studies that arrived at erroneous conclusions, due to errors in experimental designs. In this regard, it should be noted that in a recent publication, we noted that samples in approximately 75% of the papers resulting from searches with “schizophrenia” plus “DNA methylation” terms, data were obtained from samples of blood, saliva or other fluids, while the rest of the data were obtained from brain samples [2].

As we previously mentioned, mental disorders are influenced by a set of several genetic and environmental factors that genetics alone cannot explain. We believe that integration of the different epigenetic markers could explain some aspects of schizophrenia pathophysiology or, at least, could help us to define some variables that may contribute to its onset and/or development, and use this knowledge for new therapeutic approximations. In this respect, it is necessary to more closely analyze the role of interneurons on the subcortical-cortical circuitry that underlie controlled and automatic human information processing in healthy and schizophrenic human brains, and how different epigenetic marks impact on its functioning and effectiveness, not limiting the study to the DNA methylation signatures. Moreover, we need to include single cells or single-cell population analysis to avoid the overlap of different epigenomes associated to each cell type. Only in this way will we be able to shed light on the complex interaction that occurs between nature and genetics to produce schizophrenia.

## Figures and Tables

**Table 1:** Summary of epigenome-wide association studies in schizophrenic brain tissue.

Main Findings (Year)	Healthy Controls (*n*)	Patients (*n*)	Reference
2929 DMPs ^1^ (including *NOS1*, *AKT1*, *SOX10*, *DTNBP1* and *PPP3CC*) (2014).	24	24	[50]
Differential expression and methylation of *PIK3R1*, *BTN3A3*, *NHLH1* and *SLC16A7* (2014).	43	39	[51]
5 top-ranked DMPs in PFC ^2^: *GSDMD*, *RASA3*, *HTR5A*, *PPFIA1*, *CACNA1G* (2014).	23	20	[52]
5 top-ranked DMPs in cerebellum: *NAV1*, *ZNF200*, *PRH2*, *NFIA1*, *COL16A1* (2014).	23	21	[52]
107 DMPs (Hypermethylation in 79 DMP in schizophrenic brains) (2014).	110	106	[53]
1550 DMPs (Consistently DMP in schizophrenic brain: upstream or in gene regions of *CERS3*, *DPPA5*, *REC8*, *PRDM9*, *LY6G5C* and *DDX43*). (2015).	24	24	[54]
139 DMPs (5 top-ranked DMPs in PFC: *NUBP1*, *RASA3*, *STK32B*, *AIG1*, *PRKCE*. 5 top-ranked DMPs in hippocampus: *HLA-DQA1*, *HCN2*, *GPC5*, *SERPINA5*, *POLRMT*). (2016).	19	3	[55]

^1^ Differentially methylated positions; ^2^ Prefrontal cortex.

## Abbreviations

GWAS	Genome-wide association study
CpG	Cytosine-Guanine dinucleotide
BDNF	Brain-derived neurotrophic factor
COMT	Catechol-*O*-Methyltransferase
mQTL	Quantitative trait loci analysis
MSP	Methylation-specific polymerase chain reaction
SNP	Single-nucleotide polymorphisms
EWAS	Epigenome-wide association study
DMPs	Differentially methylated positions
PFC	Prefrontal cortex
TSS	Transcription start site
